# Vector design for enhancing expression level and assembly of knob-into-hole based FabscFv-Fc bispecific antibodies in CHO cells

**DOI:** 10.1093/abt/tbac025

**Published:** 2022-10-13

**Authors:** Han Kee Ong, Ngan T B Nguyen, Jiawu Bi, Yuansheng Yang

**Affiliations:** Bioprocessing Technology Institute, Agency for Science, Technology and Research (A*STAR), 20 Biopolis Way, #06-01 Centros, Singapore 138668, Singapore; Bioprocessing Technology Institute, Agency for Science, Technology and Research (A*STAR), 20 Biopolis Way, #06-01 Centros, Singapore 138668, Singapore; Bioprocessing Technology Institute, Agency for Science, Technology and Research (A*STAR), 20 Biopolis Way, #06-01 Centros, Singapore 138668, Singapore; Bioprocessing Technology Institute, Agency for Science, Technology and Research (A*STAR), 20 Biopolis Way, #06-01 Centros, Singapore 138668, Singapore

**Keywords:** CHO, bispecific antibody, knob-into-hole, vector design, recombinase-mediated-cassette-exchange (RMCE)

## Abstract

**Background:**

Two-armed FabscFv-Fc is a favoured bispecific antibody (BsAb) format due to its advantages of the conventional IgG structure. Production of FabscFv-Fc requires expression of three polypeptide chains, one light chain (LC), one heavy chain (HC) and a scFv fused to the Fc (scFvFc) at optimal ratios.

**Methods:**

We designed a set of internal ribosome entry site (IRES)-mediated multi-cistronic vectors tailoring to various expression ratios of the three polypeptides to study how the chain ratios affect the FabscFv-Fc production.

**Results:**

Expression of HC and scFvFc chains at 1:1 ratio and excess LC gave the highest yield of correctly assembled product. Compared to the use of IRES and multiple promoters, using 2A peptides for co-expression of the three polypeptides gave the highest titre and correctly assembled product.

**Conclusion:**

The results obtained in this work provide insights to the impacts of hetero-chain ratios on the BsAb production.

## Teaser

Statement of Significance: Two-armed FabscFv-Fc is a favoured bispecific antibody formats but challenging to produce. This paper presents a general strategy to customize the expression ratios of polypeptide chains to produce correctly assembled FabscFv-Fc at high titres. Similar approach can also be applied to optimize the production of other formats of bispecific antibody classes.

## INTRODUCTION

Bispecific antibodies (BsAbs) have emerged as a promising class of next-generation antibody therapeutics. In contrast to the standard monoclonal antibodies, BsAbs can recognize two different epitopes simultaneously, thereby allowing them to perform a wide range of applications that cannot be achieved with their monospecific counterparts, such as blocking multiple pathogenic mediators simultaneously and redirecting effector cells to the proximity of tumour cells. More than one hundred formats of BsAbs have been developed, which can be broadly categorized into two groups, fragment-based (no Fc) and IgG-like asymmetric BsAbs [1]. The IgG-like BsAb formats are more favoured because of their desirable pharmacokinetic (PK) properties, low immunogenicity risk, high stability and retained Fc receptor affinity [2, 3]. However, the production process for IgG-like asymmetric BsAbs are challenging due to promiscuous pairing of different antibody heavy chains (HCs) and light chains (LCs).

IgG-like asymmetric BsAbs usually consist of three to four polypeptide chains, which are often engineered to promote cognate chain pairing through framework engineering. For instance, correct LC/HC pairing can be achieved by using orthogonal Fab interface or CrossMab (one Fab with a CH1 domain switched with the partner CL domain) [4]. Alternatively, LC/HC mispairing can be overcome by using a common LC [5] or replacing Fab with single-chain fragment of variable region (scFv). Meanwhile, many engineering strategies have been developed to enhance the HC heterodimerization based on electrostatic or steric complementarity [6]. Among them, the knob-into-hole (KIH) technology [7], which involves engineering CH3 domains to create either a “knob” or a “hole” in each HC is the most successful and widely used in the industry. Faricimab, a BsAb that was developed using the KIH technology, has just been approved by FDA for treatment of wet age-related macular degeneration and diabetic macular edema [8]. Despite the effectiveness of these engineering strategies, suboptimal relative expression levels of different polypeptides of an IgG-like asymmetric BsAb still result in incorrect chain pairing and difficult-to-remove product-related contaminants. Kreudenstein and colleagues have demonstrated that for BsAbs with improved KIH heterodimeric specificity, stably transfected cells containing non-ideal chain ratios still produced large proportion of incorrectly paired species [9]. The ideal chain ratio to support high productivity and efficient assembly of IgG-like asymmetric BsAbs may not be stoichiometric and may vary depending on the molecule format. In a study of an IgG-like asymmetric BsAb consisting of a common LC and two hetero-HCs, Von Kreudenstein *et al*. found out that expressing the common LC, HC1 and HC2 at 3:1:1 ratio was optimal for efficient assembly of the correct product [9]. In another study of four-chain IgG-like asymmetric BsAbs, Rajendra *et al.* [10] revealed that the optimum ratios for expression and pairing of four distinct BsAbs were different from one another. Thus, it is critical to develop strategies which allow efficient optimization of the relative expression levels of multiple chains in specific BsAbs for obtaining maximized yield and purity.

Chinese hamster ovary (CHO) cells are the dominant host cells for producing therapeutic antibodies due to their advantages of robust cell growth and capability of performing proper post-translational modifications [11]. Studies to optimize the relative expressions of multiple chains for enhancing titre and assembly of BsAbs have been done in CHO cells in transient transfections [9, 10, 12, 13]. Co-expression of multiple chains is often achieved by co-transfecting CHO cells with multiple plasmids, with one plasmid expressing one chain. In these studies, multi-chain relative expressions were adjusted by manipulating the relative amount of the co-transfected plasmids [10, 12, 13]. However, this strategy cannot be applied in generation of commercial cell lines that need to be stably transfected. When multiple plasmids are co-transfected into mammalian cells, they would randomly integrate into the chromosome, resulting in high variations in transgene copy numbers and insertion sites across different cells [14–16]. Therefore, no meaningful correlation between the transgenes’ expression levels and optimal production of a given class of complex BsAbs could be determined. Targeted integration, which entails the insertion of transgenes into a pre-determined locus in host genome, can overcome this issue [17]. As all stably transfected cells express transgenes from the same genomic loci, the intracellular polypeptide levels are better correlated with the vector designs including gene dosage, configuration and other transcription/translation-modifying elements.

Besides integration sites, the expression level of genes are also determined by the DNA regulatory elements applied on them. Co-expression of multiple genes from a single vector can be achieved by using multiple promoters (MP), internal ribosome entry site (IRES) or 2A peptides. Arrangement of MP in one vector can result in transcriptional interference, where an active transcriptional unit suppresses the expression of other units [18]. As such, changing expression of one gene by using different strength promoters may affect the expression of other genes and thus making it difficult for obtaining defined expression ratios for different genes. In contrast to using MP, IRES has advantage of enabling expression of multiple genes in one transcript without transcriptional interference [19, 20]. Moreover, expression of each single gene could be modulated independently by using different strengths of IRES elements without affecting the expression of other genes, thus providing a powerful tool for studying the impact of various ratios of different polypeptide chains on BsAb production [21]. However, IRES-driven gene has low translational efficiency which may limit the maximal expression level that can be achieved for a gene. This issue could be overcome by using 2A peptides, which express multiple genes in one single open reading frame (ORF) and give equal amounts of co-expressed proteins, thus give high expression levels of all genes [22, 23]. The drawback of using 2A peptides for co-expressing multiple genes is that their relative expression is fixed at equal amounts and cannot be modulated.

Two-armed FabscFv-Fc is one of the most favoured asymmetric IgG-like BsAb formats due to its advantages of the conventional IgG structure. Currently 18 molecules that are developed using the FabscFv-Fc format are in clinical trials [24]. Production of FabscFv-Fc requires expression of three polypeptide chains: one light chain (LC), one heavy chain (HC) and a scFv linked to the Fc (scFvFc). The use of Fab and scFv in the two arms targeting different antigens respectively overcomes the LC mispairing issue, while efficient heterodimeric HC pairing can be obtained through the KIH technology. In this work, we aimed to study how the varied expression ratios of different polypeptides affect the FabscFv-Fc production. We designed one set of IRES-mediated multi-cistronic vectors and stably expressed them in CHO cells via recombinase-mediated-cassette-exchange (RMCE). Each multi-cistronic vector was designed to have varied expression levels of the different polypeptides by using synthetic upstream open reading frames (uORFs) and different strengths of IRES mutants [21, 25]. The impact of varying chain expression ratios on the yield of correctly assembled antibody product was studied. Subsequently, the use of IRES, 2A peptides and MP was evaluated to identify the optimal vector design for producing FabscFv-Fc.

## MATERIALS AND METHODS

### Construct of landing pad and targeting vectors

The protocols for construction of landing pad and targeting vectors were described in Supplementary Materials. The DNA sequences of different synthetic uORF used for suppression of LC expression were listed in Supplementary Table 1 available at ABT Online.

### Generation of stably transfected CHO cell pools and BsAb production

**Figure 1:**
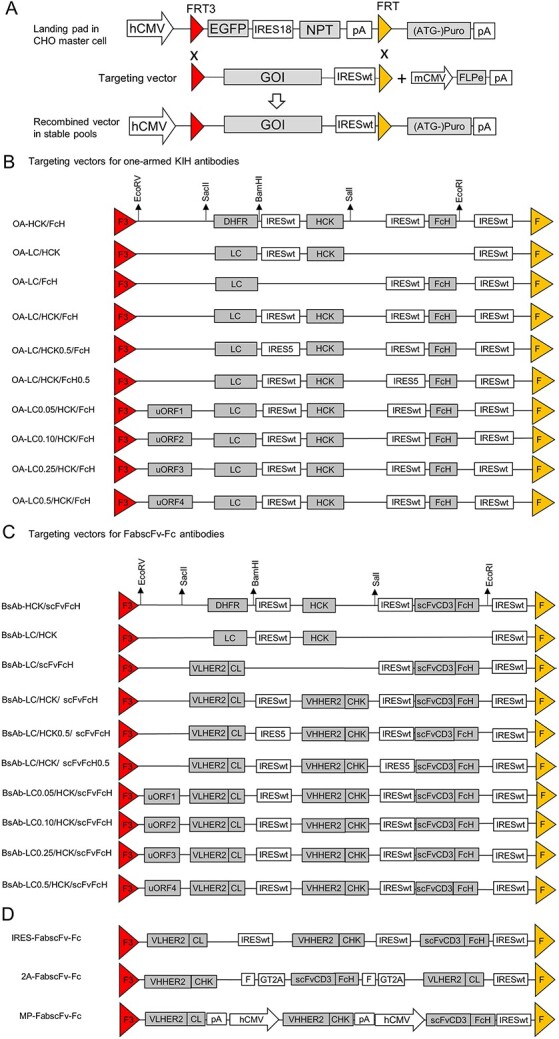
Overview of RMCE strategy and the targeting vector designs for modulating the expression ratios of LC:HCK:FcH (or scFv-FcH). (A) Schematic representation of RMCE. (B) Schematic representation of targeting vectors expressing one-armed KIH antibodies. (C) Schematic representation of targeting vectors expressing 2-armed FabscFv-Fc bispecific antibodies. (D) Schematic presentation of targeting vectors that used IRES, 2A peptides and MP for co-expression of the three polypeptides of FabscFv-Fc BsAb. hCMV, human cytomegalovirus major immediate-early gene enhancer and promoter; mCMV, murine CMV enhancer and promoter; Flpe, enhanced flippase; EGFP, cDNA encoding enhanced green fluorescence protein; NPT, neomycin phosphotransferase gene; (ATG-)Pur, puromycin resistant gene with ATG-start codon removed; FRT and FRT3, wild-type and mutated flippase recognition target; pA, SV40 polyadenylation signal; GOI, genes of interest; IRESwt, EMCV IRES; IRES5, mutated EMCV IRES variants; DHFR; dihydrofolate reductase cDNA; LC, light chain cDNA; HCK, cDNA encoding heavy chain with CH3 engineered to form a knob; FcH, cDNA encoding Fc with CH3 engineered to form a hole; scFv, cDNA encoding single chain fragment variable region; uORF, upstream open reading frame; F, furin recognition site; GT2A, GSG linker and 2A peptide derived from Thosea asigna virus.

The stably transfected CHO cell pools expressing different targeting vectors were generated via RMCE (Fig. 1A). They were characterized for growth and titre in 14-day fed-batch cultures in 50 tubespins (TPP) in the humidified Kuhner shaker (Adolf Kühner AG). Cell density, viability and antibody titre were monitored at day 3, 5, 7, 9, 11 and 14 using the Vi-Cell XR viability analyzer (Beckman Coulter) and IMMAGE 800 immunochemistry system (Beckman Coulter), respectively. The detailed protocols for generation and characterization of stable pools were described in Supplementary Materials.

### Protein a purification and size-exclusion chromatography analysis

Culture supernatants from fed-batch cultures were collected at day 14. They were purified by protein A followed by size-exclusion chromatography (SEC) analysis. The percentage of high molecular weight (HMW) and low molecular weight (LMW) species in each sample was quantified based on the total peak areas that were eluted before and after the monomeric peak, respectively. The detailed protocols were described in Supplementary Materials.

### SDS-PAGE

The purified samples were subjected to SDS-PAGE, followed by Coomassie Blue staining to assess the presence of different species in a sample. 2–3 μg of each sample were resolved on the NuPAGE™ 4–12% Bis-Tris Protein Gels (Thermo Fisher Scientific) in 3-(N-Morpholino)-Propanesulfonic Acid (MOPS) buffer following manufacturer’s protocol under both reducing and non-reducing conditions. The gels were then incubated in the fixing buffer (50% Methanol and 10% Acetic acid) for 10 min, and then followed by 10-min staining in 0.1% Coomassie blue staining solution on an orbital shaker. De-staining was performed using 30% ethanol until the background is clear. Gel photos were taken with the ChemiDoc Imaging System (Biorad).

### Western blot

Western blot was performed to detect the intracellular polypeptides in stably transfected pools. Cell lysates (5 μg each) were separated in SDS-PAGE followed by dry transfer onto a Hydrophilic polyvinylidene fluoride (PVDF) member. Membranes were blocked for 1-h followed by overnight incubation with corresponding primary antibodies. The detailed protocol was described in Supplementary Materials.

### Genomic DNA analysis

Genomic DNA (gDNA) was extracted and subjected to two junction PCRs to confirm the successful cassette exchange after recombinase-medidated-cassette-exchange (RMCE) in each stable cell pool. The detailed protocols and a list of primers used for junction polymerase chain reactions (PCRs) (Supplementary Table 2 available at ABT Online) were provided in Supplementary Materials.

### qRT-PCR analysis of mRNA levels

Total RNA was extracted, followed by cDNA synthesis. The synthesized cDNA was used as templates for qRT-PCR to analyze the relative abundance of LC, HCK and scFvFcH transcripts in stable cell pools generated using IRES, 2A peptides or MP for expressing FabscFv-Fc antibodies. The detailed protocols and a list primer used for qRT-PCR (Supplementary Table 3 available at ABT Online) were provided in Supplementary Materials.

## RESULTS

### Strategy for modulating expression levels of multiple polypeptide chains in stably transfected CHO cell pools

To study the impact of various ratios of different chains on the KIH-based antibody production, we stably expressed a set of vectors tailoring different expression levels of individual polypeptides in the CHO master cell line (MCL) via RMCE (Fig. 1). The MCL was generated via stable transfection of a landing pad vector into the parental CHO K1 cells (Fig. 1A). The landing pad vector expressed the enhanced green fluorescent protein (EGFP) and neomycin phosphotransferase (NPT) in one transcript by using a mutated encephalomyocarditis virus (EMCV) IRES variant (IRES18) [21]. Transcription of the EGFP and NPT genes were driven by a human cytomegalovirus major immediate-early gene enhancer and promoter (hCMV) and terminated by a SV40 polyadenylation signal (pA). A pair of heterospecific flippase (FLP) recognition targets, FRT3 and FRT, flanked the EGFP–IRES–NPT expression cassette. An impaired puromycin resistant gene that has its ATG start codon removed ((ATG-)Puro) was placed downstream of FRT, which cannot be expressed before successful RMCE occurred. After transfection of the landing pad vector into the CHO K1 cells, the transfected cells were selected for stable transfectants by G418. The use of IRES18, which has reduced translational efficiency, ensured that only cells expressing NPT from highly active genomic sites were able to survive the G418 selection. The MCL, which provided stable and high-level gene expression from an active genomic site, was then identified among the G418-surviving clones. To generate stably transfected pools expressing genes-of-interest (GOI) via RMCE, the MCL was co-transfected with a targeting vector carrying specific GOI and a vector expressing Flpe, followed by puromycin selection. With the promoter- and ATG-trap design, only cells with correct RMCE can activate the expression of the impaired puromycin resistant gene and survive the puromycin selection [26]. Since the surviving cells expressed GOI from the same landing pad and have similar behaviours, we were able to study the performance of the various targeting vectors in the stably transfected pools without the need for isolation of single cell-derived clones.

To express the three chains constituting KIH-based antibodies at different ratios, we designed one set of targeting vectors to carry the trastuzumab LC, engineered trastuzumab HC with a “knob” formed at CH3 domain (HCK) and an empty Fc chain (FcH) or a Fc chain fused with an anti-CD3 scFv (scFvFcH). The CH3 domain in FcH or scFvFcH was engineered to form a “hole”. The three chains were linked through multiple IRES elements (Fig. 1B and C). After integration into the landing pad in the MCL, each targeting vector would express the three polypeptide chains from one transcript under the control of the hCMV promoter. The relative expression levels of HCK and FcH or scFvFcH in each targeting vector were determined by the strengths of IRES elements applied on them. Application of the same wild-type EMCV IRES (IRESwt) on HCK and FcH or scFvFcH respectively would give same expression level of the two chains. As cap-dependent translation is more efficient than the cap-independent transfection, the LC that was directly driven by the hCMV promoter would be expressed in excess compared to the HCK and FcH or scFvFcH chains that were driven by the IRESwt. To adjust the expression levels of HCK and FcH in a targeting vector, we replaced the IRESwt on either HCK or FcH with the mutant EMCV IRES5 which had roughly 50% of translational strength of the IRESwt [21]. One set of synthetic uORFs with different strengths (Supplementary Table 3 available at ABT Online) were placed upstream of the LC translation start site to separately tune its expression to various levels without altering the transcription activity associated with hCMV promoter and the subsequent IRES-mediated transgene expression [25]. The numbers tagged to the polypeptide chains in different antibodies indicated the strength of the regulatory elements applied on the polypeptide chains. For instance, OA-LC/HCK/FcH0.5 means that application of IRES5 on FcH would reduce its expression to 0.5-fold of that driven by the IRESwt. Similarly, OA-LC0.10/HCK/FcH means application of uORF on LC would reduce its expression to 0.10-fold of the LC driven by the optimal Kozak sequence, e.g. GCCACC.

### Impact of various ratios of LC:HCK:FcH on the efficiency of heterodimeric HC pairing

**Figure 2:**
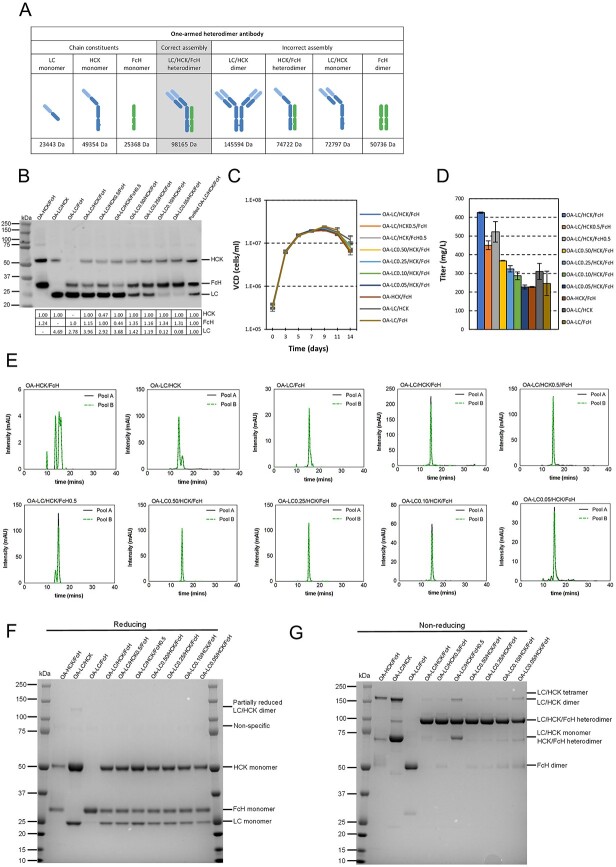
Expression and assembly of OA KIH antibodies at various expression ratios of LC:HCK:FcH. Ten vectors controlling different LC:HCK:FcH ratios were designed. Two stable cell pools were independently generated by RMCE for each vector. The abundance of intracellular polypeptides of LC, HCK and FcH in each stable pool was analyzed by western blot. The stable cell pools were characterized in 14-day fed batch cultures. The harvested culture supernatant was purified by using protein A, which was subsequently analyzed by SEC and in SDS-PAGE gel under reducing and non-reducing conditions. (A) Illustration of chain constituents, correct and incorrect assembled forms of one-armed KIH antibodies. (B) Western blot analysis of intracellular polypeptides of LC, HCK and FcH under reducing conditions. (C) VCD of the stable cell pools plotted over 14-day fed batch. (D) Titres of the stable pools at day 14 in fed-batch cultures. Each point represents the average and standard deviation of the results measured from two stable cell pools. (E) SEC chromatograms of protein A purified antibodies. Purified antibodies resolved by SDS-PAGE under (F) reducing and (G) non-reducing conditions, followed by staining with Coomassie blue. MW was indicated in kDa.

Since knob/hole heterodimerization is critical for proper assembly of KIH-based bispecific antibodies, we set out to study the impact of varying the knob to hole chain ratios on HC heterodimerization. To this end, one-armed (OA) KIH antibodies consisting of three chains, LC, HCK and FcH, were used as model antibodies (Fig. 2A). The asymmetric design of one-armed KIH antibodies facilitated convenient evaluation of knob-hole assembly since the large size differences of the mis-paired knob/hole homodimers and heterodimers could be clearly distinguished in SDS-PAGE gel and SEC. The predicted MW of correctly and incorrectly assembled OA KIH antibody species were listed in Figure 2A. A panel of seven combinatorial expression of LC, HCK and FcH genes in various ratios was constructed into the targeting vectors together with three control vectors, in which only two out of three chains were expressed (Fig. 1B). Thereafter, 20 stable cell pools, two for each vector design, expressing various ratios of LC, HCK and FcH genes were generated via RMCE (Fig. 1A). The integration of targeting vectors into the landing pad in MCL were first verified by PCRs using specific primer pairs (Supplementary Table 2 available at ABT Online). The 5′ and 3′ junction PCR amplicons of the expected sizes were detected, indicating correct RMCE happened in these stably transfected pools (Supplementary Fig. 1 available at ABT Online).

Western blot analysis of the intracellular polypeptides in these stable cell pools were then performed using antibodies against the LC and Fc regions. The relative abundance of different chains in each sample was quantified by measuring the band intensity (Fig. 2B). A control sample, the purified OA-LC/HCK/FcK antibody in which the ratio of the three chains should be at 1:1:1, was included to normalize the differences in band intensities that were brought by the variations in binding affinities of the detection antibodies against the LC, HCK and FcH polypeptides. The relative abundance of HCK was designated as 1.00 in all samples except for OA-LC/FcH and OA-LC/HCK0.5/FcH which did not contain HCK or the HCK was not under the control of IRESwt and had reduced expression. The relative abundances of LC and FcH in each sample were then calculated as the ratio of LC to HCK or FcH to HCK respectively, which were further normalized to the corresponding ratios of LC to HCK or FcH to HCK of the control sample. For OA-LC/FcH and OA-LC/HCK0.5/FcH, the relative abundance of FcH was designated as 1.00. Similarly, the relative abundances of LC and HCK in these two samples were calculated as the ratios of LC to FcH or HCK to FcH respectively, which were further normalized to the corresponding ratios of the control sample. As expected, the relative abundance of HCK and FcH were comparable (around 1:1 ratio) in the control pool OA-HCK/FcH and pools in which the expression of the two chains were both driven by IRESwt. Application of IRES5, which had half strength of the IRESwt, on either HCK or FcH effectively reduced their relative abundance to 0.47 and 0.44, respectively. In the control pool OA-LC/HCK and OA-LC/FcH and pools in which LC expression was not suppressed by uORF, the relative abundances of LC polypeptides were ranging from 2.92 to 4.69. The variation in LC abundance in these samples could be due to the experimental variation resulted from the weak signal for LC detection. Expression of LC in excess in these samples was expected as the LC gene was driven by the hCMV promoter, which translation was more efficient than HCK or FcH driven by IRESwt or IRES5. In the pools in which LC expression was under the control of different uORFs, the relative abundance of LC polypeptides gradually decreased corresponding to the strengths of different uORFs [25], while the relative abundance of HCK and FcH polypeptides in these pools remained unchanged or slightly increased. The magnitude of reduction of the LC polypeptide abundance did not match the expected levels that were supposed to be induced by the corresponding uORFs based on their strengths. For instance, the relative abundance of LC polypeptide in the sample OA-LC/HCK/FcH was at 3.96. The OA-LC0.5/HCK/FcH was expected to have relative abundance of LC polypeptides at 1.98. However, the LC relative abundances determined by western blot in this sample was 1.42. Besides experimental variation, another possible reason that caused the inconsistency is that the strengths of uORF varies depending on the specific proteins.

The stably transfected pools were characterized in 14-day fed-batch cultures to examine the effects of different LC:HCK:FcH ratios on the expression level and assembly of one-armed KIH antibodies. Varying the ratios of the three polypeptide chains by reducing expression of LC, HCK or FcH had no impact on cell growth as indicated by the similar viable cell density (VCD) over the 14-day fed-batch culture period across different stable pools (Fig. 2C). However, the titres of OA antibodies were significantly reduced with the reduced expression of different polypeptide chains (Fig. 2D). The highest antibody titres were obtained in the wild-type OA-LC/HCK/FcH stable pools. In contrast, reducing either the HCK or FcH expression by approximately 50% in the OA-LC/HCK0.5/FcH and OA-LC/HCK/FcH0.5 stable pools resulted in reduced antibody titres by roughly 20%. Similarly, reducing LC expression without changing HCK and FcH expression also resulted in decreased antibodies titres. Compared to the wild-type OA-LC/HCK/FcH pools which had the relative abundance of intracellular LC polypeptides at 3.96 (Fig. 2B), reducing the relative abundance of LC polypeptide to 1.42 in the OA-LC0.50/HCK/FcH pools resulted in reduced antibody titre by 40% (Fig. 2D). Further reducing the LC expression resulted in gradually reduced antibody titres. Interestingly, in the OA-LC0.05/HCK/FcH stable pool that had relative abundance of LC polypeptides at 0.08, its antibody titre still maintained more than 30% of the wild-type OA-LC/HCK/FcH pool. In fact, titres greater more than 30% of the wild-type OA-LC/HCK/FcH pools were also detected in the three control pools OA-HCK/FcH, OA-LC/HCK and OA-LC/FcH, which were missing either LC, FcH or HCK component.

The culture supernatants harvested from different stable cell pools were purified by Protein A to obtain materials for chain assembly analysis. SEC was used to examine the product purity profile by distinguishing the size difference of species resulted from correct and incorrect chain assembly. When expression of HCK and FcH polypeptides were roughly at 1:1 ratio, more than 90% of the product was correctly assembled with minimal aggregate or incompletely unassembled fragments (Fig. 2E). Reducing FcH expression in the OA-LC/HCK/FcH0.5 pools increased the level of aggregate to 20%, suggesting inefficient pairing of knob and hole chains (Table 1). In contrast, little changes were observed in the SEC profile for the purified antibodies produced in the OA-LC/HCK0.5/FcH pools, although the HCK: FcH ratio was also disturbed in these stable pools by reducing the HCK expression (Fig. 2E). Similarly, moderately reducing LC expression did not strongly impair the knob and hole heterodimerization as seen in OA-LC0.50/HCK/FcH and OA-LC0.25/HCK/FcH pools. However, strong suppression of LC expression increased accumulation of aggregates and fragments in the OA-LC0.10/HCK/FcH pools (3% of aggregates) and OA-LC0.05/HCK/FcH pools (9% of aggregates and 4% of LMW fragments) (Table 1). Together, these findings suggested that controlling expression of HCK and FcH at 1:1 ratio and LC at excess amount were optimal for obtaining correct chain assembly as compared to other ratios. Following SEC analysis, purified antibodies were resolved on reducing and non-reducing SDS-PAGE gel to further characterize the structural integrity and different species formed by LC, HCK and FcH assembly. Under reducing conditions, distinct LC, HCK and FcH monomers were resolved at the predicted MWs across all expression ratios (Fig. 2F), confirming the intact structure of each subunit chain. A weak band corresponding to LC/HCK dimers was also detected in OA-LC/HCK pools, potentially due to incompletely reduction. In non-reducing conditions, similar prominent bands were detected at roughly 100 kDa in all LC:HCK:FcH ratio combinations, corresponding to the size of the correctly assembled one-armed KIH antibody (Fig. 2G). A 100-kDa band was also detected in the OA-LC/HCK control vector, which could be HCK dimers as this vector does not contain FcH chain. Several wrongly assembled products were also detected at the MW of 150, 75 and 50 kDa. The bands at 150 kDa corresponded to the mis-paired LC/HCK homodimers, which were confirmed in the product expressed from the OA-LC/HCK control vector. 75-kDa bands could be either LC/HCK monomers or HCK/FcH heterodimers, which cannot be discriminated due to their similar MWs. These species were prominent in products expressed from the OA-LC/HCK and OA-HCK/FcH control vectors, respectively. In addition, a weak band at above 150 kDa was detected only in the OA-LC/HCK control vector corresponded to the mis-paired LC/HCK tetramers. Lastly, the bands at 50 kDa corresponded to the FcH homodimer, which were found in product expressed from the OA-LC/FcH control vector. The proportion of these undesired by-products were largely small as compared to the dominant band LC/HCK/FcH heterodimer in most products except those expressed from the LC:HCK:FcH0.5 vector. However, the intensity of by-product bands started to increase as the LC expression reduced. These data further confirmed that expression of knob and hole chains at 1:1 ratio and excess LC yielded most efficient assembly of heteromeric HC pairing and minimized the unwanted by-products.

**Table 1:** Relative distribution of different species detected in SEC profiles of the protein-A purified materials produced in stable cell pools generated using different vectors

Expression ratio	Purity %		
	Aggregate	LC/HCK/FcH (or scFvFcH) heterodimer	Fragment
**One-armed KIH antibody**			
OA-HCK/FcH	98.30 ± 2.40	0	1.70 ± 2.40
OA-LC/CHK	77.52 ± 0.26	0	22.5 ± 0.25
OA-LC/FcH	8.73 ± 2.93	0	91.28 ± 2.39
OA-LC/HCK/FcH	1.75 ± 0.23	98.25 ± 0.24	0
OA-LC/HCK0.5/FcH	1.20 ± 0.16	98.80 ± 0.16	0
OA-LC/HCK/FcH0.5	20.91 ± 3.71	79.09 ± 3.71	0
OA-LC0.50/HCK/FcH	0.82 ± 0.02	99.19 ± 0.02	0
OA-LC0.25/HCK/FcH	1.13 ± 0.04	98.88 ± 0.05	0
OA-LC0.10/HCK/FcH	3.23 ± 0.71	96.77 ± 0.72	0
OA-LC0.05/HCK/FcH	9.08 ± 0.01	86.59 ± 0.23	4.34 ± 0.25
**FabscFv-Fc bispecific antibody**			
BsAb-HCK/FcH	44.60 ± 1.62	0	55.42 ± 1.62
BsAb -LC/CHK	78.03 ± 0.28	0	21.97 ± 0.20
BsAb -LC/FcH	7.35 ± 0.76	0	92.65 ± 2.17
BsAb -LC/HCK/FcH	10.78 ± 0.47	89.02 ± 0.18	0.20 ± 0.28
BsAb -LC/HCK0.5/FcH	11.96 ± 0.66	87.84 ± 0.38	0.21 ± 0.29
BsAb-LC/HCK/FcH0.5	21.80 ± 0.39	75.38 ± 0.66	2.82 ± 0.28
BsAb-LC0.50/HCK/FcH	11.44 ± 0.37	88.27 ± 0.35	0.29 ± 0.03
BsAb-LC0.25/HCK/FcH	11.49 ± 0.57	88.25 ± 0.56	0.26 ± 0.01
BsAb-LC0.10/HCK/FcH	13.29 ± 1.38	86.49 ± 1.24	0.32 ± 0.13
BsAb-LC0.05/HCK/FcH	17.33 ± 1.57	68.78 ± 1.12	13.99 ± 0.44
**IRES, 2A peptides and multiple-promoters**			
IRES-FabscFv-Fc	9.91 ± 0.03	90.90 ± 0.03	0
2A-FabscFv-Fc	9.94 ± 0.45	90.06 ± 0.45	0
MP-FabscFv-Fc	6.49 ± 0.36	0	93.527 ± 0.35

### Optimal ratios of LC:HCK:scFvFcH for obtaining high expression and efficient assembly of 2-armed FabscFv-Fc bispecific antibodies

**Figure 3:**
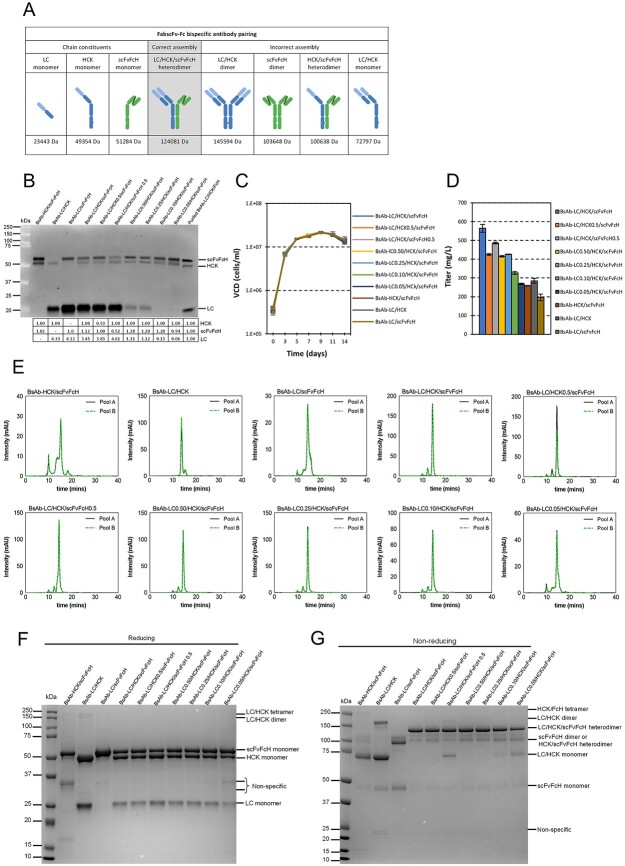
Expression and assembly of FabscFv-Fc bispecific antibodies at various expression ratios of LC:HCK:scFvFcH. Ten vectors controlling different LC:HCK:scFvFcH ratios were designed. Two stable cell pools were independently generated by RMCE for each vector. The intracellular polypeptides of LC, HCK and scFv-FcH in each stable pool was analyzed by western blot. The stable pools were characterized in 14-day fed-batch cultures. The harvested culture supernatant was purified by using protein A, which was subsequently analyzed by SEC and in SDS-PAGE under reducing and non-reducing conditions. (A) Illustration of chain constituents, correct, and incorrect assembled forms of FabscFvFcH bispecific antibodies. (B) Western blot analysis of the intracellular polypeptides under reducing conditions. (C) VCD of the stable cell pools plotted over 14-day fed-batch cultures. (D) Titres of the stable pools at day 14 in fed-batch cultures. Each point represents the average and standard deviation of the results measured from two stable cell pools. (E) SEC chromatograms of protein A purified antibodies. Purified antibodies resolved by SDS-PAGE under (F) reducing and (G) non-reducing conditions, followed by staining with Coomassie blue. MW was indicated in kDa.

Upon understanding how different ratios of subunits influenced the assembly and expression of HC heterodimerization, we further studied the optimal expression ratios for producing the 2-armed FabscFv-Fc BsAb. In contrast to the OA KIH antibodies, FabscFv-Fc had the FcH chain fused with an anti-CD3 scFv (Fig. 3A). Such fusion polypeptides could alter the overall expression and assembly of BsAb due to unpredictable changes in protein folding. In addition, the increased structural complexity could also affect the assembly, potentially resulting in undesired by-products as illustrated in Figure 3A. Following the design for OA KIH antibodies, 10 IRES-mediated multi-cistronic vectors expressing various ratios of LC:HCK:scFvFcH were designed and stably expressed in the MCL via RMCE (Fig. 1A and C). Junction PCRs confirmed the successful integration of the targeting vectors into the landing pad in the MCL (Supplementary Fig. 2 available at ABT Online). The relative expression of the three chains in different stable pools were verified via western blot. Consistent with the results for the one-armed KIH antibodies, applying IRES5 on either HCK and scFv-FcH reduced their relative abundance to roughly 0.50 (Fig. 3B). Similarly, the abundance of intracellular LC polypeptides were reduced in a controlled manner by placing various uORFs upstream of the LC translation start sites (Fig. 3B). Same as observed for one-armed antibodies, the relative abundance of the LC polypeptides for the LC0.5 and LC0.25 did not reflect the strengths of uORFs applied on them, giving 1.11 and 1.12, respectively. The inconsistency could be resulted from the experimental variation and/or the protein specific effect of uORF’s strength. We then examined the cell growth and titre of these stably transfected pools in 14-day fed-batch cultures. Similar to the findings as those for the one-armed KIH antibodies (Fig. 2C), different stable pools expressing various ratios of LC:HCK:scFvFcH exhibited similar VCD profiles over the 14-day fed-batch period (Fig. 3C). The wild-type stable pools BsAb-LC/HCK/scFvFcH gave the highest BsAb titres and reducing expression of different polypeptides in other pools resulted in reduced titres (Fig. 3D).

Chain assembly efficiency was studied using SEC and SDS-PAGE under reducing and non-reducing conditions. The products expressed from the three control vectors, which contained genes encoding two of the three chains, had high levels of aggregates and fragments. In contrast, the vectors expressing all three chains and HCK and scFvFcH at 1:1 ratio produced products with similar SEC profiles (Fig. 3E), containing up to 88% of full BsAb (LC/HCK/scFvFcH heterodimer), roughly 12% of high MW aggregates and little incompletely assembled chains (Table 1). Tilting the ratios towards lesser expression of scFvFcH or LC slightly increased the accumulation of both aggregates (16–22%) and incompletely assembled fragments (2–14%) as seen in stable pools of LC/HCK/scFvFcH0.5 and LC0.05/HCK/scFvFcH. In the reducing SDS-PAGE gel, distinct protein bands, corresponding to the correct MW of LC, HCK and scFvFcH subunits, were observed across all ratios, suggesting the structure integrity of each polypeptide (Fig. 3F). Weak bands corresponding to partially reduced products like LC/HCK tetramers and dimers were also detected in the control vector BsAb-LC/HCK. Under non-reducing condition, the most intense bands were detected at the anticipated molecular size of LC/HCK/scFvFcH heterodimer across all ratios of LC:HCK:scFvFcH (Fig. 3G). Unwanted by-products, corresponding to the size of scFvFcH dimers, HCK/scFvFcH heterodimers and LC/HCK monomers were also observed but least noticeable in the BsAb-LC/HCK/scFvFcH stable pools. Together with the titre analysis, we concluded that expressing HCK and scFvFcH at equal amount and excess LC was optimal for producing FabscFv-Fc with efficient assembly and high titre.

### Comparison of IRES, multiple promoters and 2A peptides for expressing FabscFv-Fc bispecific antibodies

**Figure 4:**
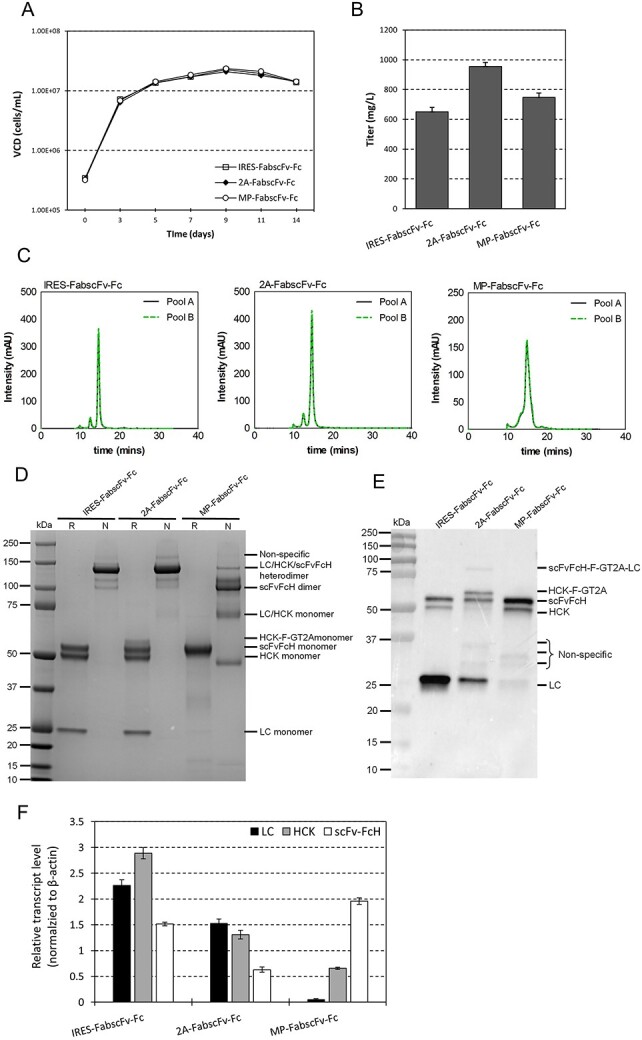
Expression and assembly of FabscFv-Fc bispecific antibodies produced using IRES-, 2A peptide (2A)- and MP-vector. Two stable cell pools were independently generated by RMCE for each vector. The intracellular polypeptides of LC, HCK and scFv-FcH in each stable pool was analyzed by western blot. The stable cell pools were characterized in 14-day fed-batch cultures. The harvested culture supernatant was purified by using protein A, which was subsequently analyzed by SEC and in SDS-PAGE gel under reducing and non-reducing conditions. (A) VCD of the stable cell pools plotted over 14-day fed-batch cultures. (B) Titres of the stable pools at day 14 of fed batch cultures. Each point represents the average and standard deviation of the results measured from two stable cell pools. (C) SEC chromatograms of protein A purified antibodies. Purified antibodies resolved by SDS-PAGE under (D) non-reducing and reducing conditions, followed by staining with Coomassie blue. MW was indicated in kDa. (E) Western blot analysis of the intracellular polypeptides. R, reducing condition. N, non-reducing condition. (F) qRT-PCR analysis for LC, HCK and scFv-FcH transcript levels in stable pools generated using 2A peptide, IRES and MP vectors. The transcript level of each gene was normalized to the internal control β-actin.

The LC/HCK/scFvFcH stable pools, which expressed the HCK and scFv-FcH at 1:1 ratio and LC in excess by using multiple IRES, gave the highest titre and most efficient assembly of FabscFv-Fc (Fig. 3E and G). Reducing LC expression did not hinder correct heteromeric HC chain assembly until the LC amount reached critically low levels (LC0.05). Despite IRES being effective for controlling the relative expression of HCK and scFv-FcH at equal amount, its low translational efficiency may limit the maximal expression titre that can be achieved by a vector [19, 27]. It has reported that co-expression of multiple chains in a single vector using MP obtained high titres of BsAbs in stably transfected CHO cells [10]. We previously demonstrated that co-expressing the LC and HC of monoclonal antibodies in CHO cells by using 2A peptides gave higher titres than the use of IRES [20] As such, we set out to compare IRES, MP and 2A peptide in expressing FabscFv-Fc bispecific antibodies. In the MP targeting vector, same hCMV promoters were used to drive the expression of LC, HCK and scFvFcH, respectively (Fig. 1C). In the 2A targeting vector, the three genes were linked through two F-GT2A, which were the combination of a furin recognition site (F), GSG linker (G) and a 2A peptide derived from Thosea asigna virus (T2A). The furin recognition site was placed in front of GT2A sequence to remove the remaining 2A residues that were attached to the upstream polypeptide after 2A cleavage. Among different 2A peptides, F-GT2A has been demonstrated to be the most efficient for obtaining high expression and correct assembly of monoclonal antibodies [22]. Stably transfected pools expressing FabscFv-Fc BsAb using the three vector systems were generated via RMCE, followed by characterization of growth and productivity in 14-day fed-batch cultures. Junction PCRs confirmed the successful integration of the targeting vectors into the landing pad in the MCL (Supplementary Fig. 3 available at ABT Online). Although all stable pools displayed very uniform growth profile throughout fed-batch culture (Fig. 4A), the stable pools using 2A peptide produced the highest titre, which was 40% higher than that in the IRES pools. The MP pools also had 15% of increment in titre compared to the IRES pools (Fig. 4B). The SEC profiles of FabscFv-Fc produced in 2A pools exhibited similar purity pattern compared to those produced in the IRES pools (Fig. 4C). 90% of product was the correctly assembled LC/HCK/scFvFcH full BsAb (Table 1). In contrast, the products from the MP pools contained up to 93% of unassembled chains (Fig. 4C, Table 1).

Under reducing condition, the purified products expressed from IRES and 2A targeting vectors showed three distinct bands corresponding to the correct MW of the three peptides LC, HCK and scFv-FcH, respectively (Fig. 4D). Majority of them were assembled into correct LC/HCK/scFvFcH heterodimer forms, as indicated by prominent protein bands at 130 kDa in the non-reducing gel (Fig. 4D). Under reducing conditions, we observed a weak band with size slightly larger than the bands corresponding to the correct scFvFcH and HCK monomer in the purified product expressed from the 2A targeting vector (Fig. 4D). Western blot analysis of the intracellular polypeptides expressed from the 2A vector also identified a band which size was greater than the HCK polypeptide (Fig. 4E). These two bands may be the HCK polypeptides attached with 2A residues (HCK-F-GT2A) resulted from incomplete cleavage at the furin recognition site. Our previous study indicated that antibodies attached with 2A residues can be removed from the correct product by using chromatin-directed clarification step [22] A very weak band at above 75 kDa was also detected in product expressed from the 2A peptide vector, which could be a fusion protein of scFvFcH-F-GT2A-LC. However, this polypeptide was not likely to secret out of the cells as it cannot fold properly. Analysis of the purified product expressed from the MP targeting vector in the reducing SDS-PAGE indicated that the LC and HCK polypeptides were not detectable at their anticipated sizes. Meanwhile, many wrongly assembled molecules were detected in the non-reducing gel, indicating inefficient chain assembly. The western blot result indicated that the LC polypeptides were barely expressed in MP pools (Fig. 4E), which explained for the poor chain assembly observed in Figure 4D. To understand why the MP vector had low expression of LC polypeptides as well as why the 2A peptide vector gave higher BsAb titre than the IRES vector, we performed qRT-PCR analysis of the LC, HCK and scFvFcH mRNA levels expressed from these three vectors (Fig. 4F). The LC mRNA expressed form the MP vector was barely detected, suggesting its expression was suppressed at the transcriptional level probably due to transcriptional interference. Interestingly, the IRES vector expressed more LC, HCK and scFv-Fc transcripts than the 2A vector although the latter vector produced higher BsAb titres (Fig. 4B). This observation suggested that the higher titre given by the 2A peptide vector may be due to the higher translation efficiency of the 2A linked HCK and scFvFcH than those driven by IRES.

## DISCUSSION

Many antibody framework engineering strategies, such as KIH technology, are effective to solve the multiple chains-mispairing problems. However, efficient production of correctly assembled BsAb still requires expression of their constitute chains at optimal ratios. Being able to design vectors that can customize the relative expression levels of multiple chains is advantageous for robust production of complex BsAb molecules. To study how the chain ratios affect the FabscFv-Fc production, we designed a set of IRES-mediated multi-cistronic vectors tailoring to a range of various expression ratios of their constitute chains and stably expressed them in CHO cells via targeted integration. We found out that expression of HCK and scFv-FcH chains at similar amount and LC in excess was optimal for enhancing the yield of correctly assembled FabscFv-Fc. Compared to the use of IRES and MP, using 2A peptide for co-expression of the three polypeptides gave the highest BsAb expression level and proportion of correctly assembled product. However, incomplete cleavage at furin recognition site and 2A resulted in production of some products attached with 2A residues. Further engineering of furin recognition site and 2A for enhancing cleavage efficiency is needed to overcome this issue. The results in this study shed insights into the correlation between the optimum ratios of chain expression levels and the efficiency of BsAb assembly, which could be extrapolated to the production of other KIH-based BsAbs. In addition, the vector design strategies presented here can also be applied in optimizing the production of other formats of BsAbs in which the optimal chain ratios could be different and varied depending on the specific formats and molecules.

Consistent with a previous study, we found out that equal expression of knob and hole chains was optimal for enhancing the yield of correctly assembled products for both KIH based one-armed model antibodies and 2-armed FabscFv-Fc BsAbs [3]. Deviation from the 1:1 ratio by reducing the FcH expression resulted in increased level of aggregates, while decreasing HCK expression had lesser negative effect on the product assembly (Figs 2E and  3E). It appears that excess HCK chains without enough FcH pairing partners have the propensity to aggregate. The tendency of the knob chains to precipitate at higher concentrations is expected as the mutant (T366W) is more exposed in knob monomer than the hole monomer consisting of the mutants (T366S, L368A and Y407V) [3, 5, 28]. As such, it is recommended to fuse the more complicated sequences to FcH rather than to FcK (Fc knob) for obtaining the highest expression titres [3]. Equal expression of knob and hole chains is optimal for efficient asymmetric antibody production regardless of using the empty FcH or fusion of a scFv to the FcH. Our inhouse study also indicated fusion of even more complex sequence, such as two tandem scFvs to FcH did not change the optimal ratio of knob and hole chains at 1:1 for obtaining correctly assembled product. In addition, expressing knob and hole chains at equal ratio had been confirmed optimal for production of FabscFv-Fc BsAbs in which the anti-HER2 Fab was replaced with two other Fab antibodies (unpublished results). These results suggest that equal expression of the two heterometric HCs could be optimal for production of any KIH based BsAbs.

For monoclonal antibodies (mAbs), it is recognized that expression of excess LC over HC is desirable for enhancing the expression levels and reducing aggregation [29–31]. The interaction of CH1 and the LC constant region is the prerequisite for proper HC folding. By reducing the accumulation of unfolded HC, excess LC polypeptides facilitate more efficient IgG assembly and secretion [32–34]. Regarding the role of LC in producing BsAbs, a previous study has reported that in stably transfected CHO cells, co-transfecting plasmids expressing common LC, HC1 and HC2 at 3:1:1 ratio was optimal for obtaining correctly assembled common LC IgG-like asymmetric BsAb [9]. Consistent with this study, we also found that under the condition of equal expression of knob and hole subunits, expressing LC in excess is beneficial for enhancing the yield of correctly assembled FabscFv-Fc. We speculate that an excess of LC could facilitate the proper folding of HCK and scFvFcH polypeptides and thus enhancing the efficiency of their pairing. It should be noted that expression of LC in excess may not be desirable for producing the four-chain IgG-like asymmetric BsAbs which consist of two LC, as surplus LC may increase its propensity to pair with noncognate HC [35].

## Supplementary Material

2022-08-22_R1_BsAb_vector_design_Sup_Materials_Clean_tbac025

## Data Availability

Supplementary Materials accompanying this paper is available.

## ANIMAL RESEARCH STATEMENT

No animal was used in this work.
