# Supplementary material for: Vector design for enhancing expression level and assembly of knob-into-hole based FabscFv-Fc bispecific antibodies in CHO cells
Source: Antib Ther. 2022 Oct 13;5(4):288–300. doi: 10.1093/abt/tbac025 (PMC9743168; doi:10.1093/abt/tbac025)
Supplement: 2022-08-22_R1_BsAb_vector_design_Sup_Materials_Clean_tbac025 [file 2022-08-22_r1_bsab_vector_design_sup_materials_clean_tbac025.docx]

**Supplementary Materials**

**Title**

Vector design for enhancing expression level and assembly of knob-into-hole based FabscFv-Fc bispecific antibodies in CHO cells

**Authors and affiliations**

Han Kee Ong#^1^, Ngan T.B. Nguyen #^1^, Jiawu Bi^1^, Yuansheng Yang*****^1^

^1^Bioprocessing Technology Institute, Agency for Science, Technology and Research (A*STAR), Singapore, Singapore

# Equal contribution

*****Corresponding authors:

Dr. Yuansheng Yang, Bioprocessing Technology Institute, Agency for Science, Technology and Research (A*STAR), 20 Biopolis Way, #06-01 Centros, Singapore 138668.

(yang_yuansheng@bti.a-star.edu.sg)

**Materials and methods**

**Construct of landing pad and targeting vectors**

The landing pad vector for generation of the CHO master cell line (MCL) was constructed by GenScript. The two genes coding for an enhanced green fluorescent protein (EGFP) and neomycin phosphotransferase (NPT) were linked through a mutated encephalomyocarditis virus (EMCV) internal ribosome entry site (IRES18). A human cytomegalovirus major immediate early gene enhancer and promoter (hCMV, -1143 to +824 relative to the transcriptional start site of +1, NCBI: M60321) was used to control the EGFP and NPT transcription. The expression cassette was flanked by FLP recognition target FRT3 and FRT sites as shown in Fig 1A. An impaired puromycin resistant gene lacking the start codon ((ATG-)Puro) followed by the simian virus 40 (SV40) polyadenylation signal (pA) was placed downstream of FRT. The DNA sequences of IRESwt, IRES18, FRT, FRT3, pA, and enhanced FLP recombinase (FLPe) were previously described in our previous study (1).

The two antibody formats in this study were one-armed KIH and two-armed FabscFv-Fc. One-armed KIH antibodies consist of three polypeptide chains: (1) a standard trastuzumab LC, (2) an engineered trastuzumab HC (full length) with a “knob” formed in its CH3 domain (HCK), and (3) an anti-CD3 Fc region containing a “hole” in its CH3 domain (FcH). The knob and hole variants were designed to enhance HC heterodimerization (2, 3). Two-armed FabscFv-Fc BsAb antibodies share similar structures with one-armed KIH antibodies except that the FcH fragment was fused with an anti-CD3 single chain variable fragment (scFv) through (G4S)3 linker (scFvFcH). The anti-HER2 LC, anti-HER2 HC, anti-CD3 scFv and anti-CD3 FcH cDNAs were designed based on the amino acid sequence of trastuzumab and pasotuxizumab published in the international ImMunoGeneTics information system (IMGT) respectively.

For targeting vectors expressing one-armed KIH antibody, a basic targeting vector FRT3-DHFR-IRESwt-HCK-IRESwt-FcH-IRESwt-FRT (OA-HCK/FcH) was constructed by GenScript. Five unique restriction sites were added into this vector as shown in Fig 1B to facilitate cloning of other targeting vectors expressing one-armed KIH antibodies. The DHFR cDNA sequence was replaced with LC cDNA sequence (synthesized by GenScript) using SacII and BamHI restriction (RE) sites to generate targeting vector OA-LC/HCK/FcH. Fragments containing IRES5-HCK and IRES5-FcH were also synthetized by GenScript followed by cloning into OA-LC/HCK/FcH vector using RE pair BamHI-SalI and SalI-EcoRI respectively. The four uORF1, uORF2, uORF3, and uORF4 were synthesized together with the LC gene and inserted into OA-LC/HCK/FcH plasmid using EcoRV and SacII sites. Their sequences were obtained from a previous study and listed in Supplement Table 1 (4). Two control vectors OA-LC/HCK and OA-LC/FcH were also constructed by GenScript. For targeting vectors expressing FabscFv-Fc antibodies, the first basic plasmid, BsAb-HCK/scFvFcH was derived from OA-HCK/FcH vector by replacing the FcH with scFvFcH fragment (GenScript) using SalI and EcoRI sites. Similar strategy for construction of one-armed KIH antibodies was used for construction of different targeting vectors expressing FabscFv-Fc antibodies except that the BsAb-HCK/scFvFcH plasmid was used as the basic vector. All vectors were propagated using NEB 5-alpha Competent *E. Coli* (NEB).

**Generation of stably transfected CHO cell pools and BsAb production**

The CHO MCL were co-transfected with 5 µg of an appropriate targeting vector and 5 µg of a vector expressing FLPe using Amaxa SG Cell Line 4D-Nucleofector® X Kit L (Lonza). Selection for stable cell pools were performed five days post transfection by culturing the transfection cells in maintenance media containing 20 µg/mL Puromycin (Invivogen). Selection was carried out for two weeks by passaging cells in the selection medium every 3-4 days until the cell viability reached 95% and above. 14-day fed-batch cultures were performed for each stable pool in 50 mL tubespins (TPP) in the humidified Kuhner shaker (Adolf Kühner AG) with 8% CO2 at 37°C. The volume of each fed-batch culture was 30 mL. The inoculation viable cell density (VCD) was at 3×105 cells/mL. The growth medium was protein-free and consisted of 50% of HyQ PF (GE Healthcare Life Sciences) and 50% of CD CHO (ThermoFisher) supplemented with 1g/L sodium carbonate (Sigma), 6 mM glutamine (Sigma) and 0.05% Pluronic F-68 (Thermo Fisher). 3 mL of Ex-Cell Advanced CHO Feed 1 (with glucose) (Sigma) was added into fed-batch cultures at day 5, 7, 9 and 11. In addition, 45% (w/v) D-glucose (Sigma) was supplemented to the culture accordingly to maintain the glucose concentration greater than 2 g/L. Cell density, viability and antibody titer were monitored at day 3, 5, 7, 9, 11 and 14 using the Vi-Cell XR viability analyser (Beckman Coulter) and IMMAGE 800 immunochemistry system (Beckman Coulter), respectively.

**Protein A purification and SEC**

Supernatants from fed-batch cultures were collected at day 14 followed by purification using TOYOPEARL® AF-rProtein A-650F resin (Tosoh Bioscience) packed in a Tricorn 10/50 chromatography column (Cytiva) on a GE AKTA Purifier 100 FPLC System (GE Healthcare, Uppsala, Sweden). Buffers containing 150 mM sodium chloride (NaCl) and 50mM 2-[4-(2-hydroxyethyl)piperazin-1-yl]ethanesulfonic acid (HEPES) at pH 7.0 was used to equilibrate (1.5 CV) and wash (15 CV) the column before and after sample loading respectively. Elution was performed with 100 mM acetic acid, pH 3.5, followed by neutralization with 1 M Trizma base (Sigma) to achieve pH 6.0-7.0. Eluents were further filtered through a 0.20 µm syringe filter prior to subsequent assays. The purified samples were analyzed by HPLC-SEC using a TSK_gel_ G3000SW_XL_ column (7.8mm i.d. x 30cm; Tosoh Bioscience) at a flow rate of 0.6 mL/min. The mobile phase comprises of 50 mM MES, 200 mM L-arginine, 5 mM EDTA, 0.05% sodium azide (w/w) at pH 6.5. The resultant concentrations were determined by comparing the area of the peaks observed at 280 nm UV absorbance to a calibration curve obtained using standard samples.

## Western blot

Western blot was performed to detect the intracellular expression of LC and HC in stable cell pools. Cell lysate was obtained from 10 million cells using CelLytic M reagent (Sigma) supplemented with Halt Protease and Phosphatase Inhibitor Cocktail (ThermoFisher). Protein concentration was then quantifed using DC Protein Assay (Biorad) on the Infinite 200 PRO plate reader (TECAN). Samples (5µg each) were separated using 12% Mini-PROTEAN® TGX™ Precast Protein Gels (Biorad) in Tris/Glycine/SDS buffer according to manufacturer’s protocol under reducing condition. 70 ng of purified antibodies from sample OA-LC/HCK/FcH or BsAb-LC/HCK/scFvFcH were used as an internal control. Proteins were then transferred onto a PVDF membrane using the iBlot Dry Blotting system (Life Technologies), followed by 1-hour incubation in blocking buffer (5% Blotting-Grade Blocker (Biorad) in TBS with 0.15% Tween 20 (TBST)). Membranes were probed with HRP-conjugated antibodies against human Kappa Light Chain (1:20000) (Bethyl) and human IgG-Fc Fragment (1:1500) (Bethyl) overnight at 4^o^C. After washing, chemiluminescence detection was carried out using the ECL Prime Western Blotting System (GE Healthcare) on the ChemiDoc Imaging System (Biorad) to visualize the membranes. The intensity of each band was finally measured using Image J software.

## Genomic DNA analysis

Genomic DNA (gDNA) was extracted from 5 million cells using PureLink® Genomic DNA Kits (Life Technologies) according to manufacturer’s protocol. Junction PCRs were performed to determine correct cassette exchange via RMCE in stably transfected pools. 40 ng of gDNA was used as template for PCR with Platinum SuperFi II Green PCR Master Mix (Thermo Fisher Scientific), and thermocycler conditions according to the manufacturer’s protocols. Thereafter, PCR reaction was resolved in 1% agarose gels stained with ethidium bromide followed by visualization step using InGenius Bio Imaging system. List of primers for 5’ and 3’ junction PCR and their expected band sizes are summarized in Supplementary Table 2.

**Quantitative real time PCR**

RNA was extracted from 5 million cells using RNeasy Mini Kits (Qiagen), followed by cDNA synthesis using EvoScript Universal cDNA Master (Roche) with 2.5 µg of the isolated RNA. A 10x dilution was performed on the cDNA and 2 µL of the diluted cDNA was added as the template for qRT-PCR, together with 5 µL of 2X FastStart Essential DNA Green Master (Roche), 0.5 µL each of 10 µM forward and reverse primer and 2 µL of nuclease-free water. The qRT-PCR was performed on a LightCycler® 96 Real Time PCR system (Roche) and analyzed with the LightCycler® 96 software (Roche). CHO β-actin was used as the internal control to normalize the CT value of the individual gene of interest. The list of primers used are listed in Supplementary Table 3.

**Supplementary Table 1.** List of uORFs used in this work

|  | **Name** | **Sequence (5’-3’)** |
| --- | --- | --- |
| 1 | **uORF1** | **ACC**ATGGGTTGA**TTT**ATG |
| 2 | **uORF2** | **GGG**ATGGGTTGA**TTT**ATG |
| 3 | **uORF3** | **TTT**ATGGGTTGA**GGG**ATG |
| 4 | **uORF4** | **TTT**ATGGGTTGA**TTG**ATG |

**Supplementary Table 2.** List of primers for junction PCRs

| **Cell line** | **Primer ID** | **Primer sequence (5’-3’)** | **Expected PCR size** |
| --- | --- | --- | --- |
| OA-HCK/FcH | CMV-F | CTAACAGACTGTTCCTTTCCA | 512bp |
|  | DHFR-R | GCCTGGTTGATTCATGGC |  |
|  | HC-F2 | GAGGAGCAGTACAACAGCAC | 1147bp |
|  | PUR-R | GCACCGTGGGCTTGTACT |  |
| OA-LC/HCK  (also, BsAb-LC/HCK) | CMV-F | CTAACAGACTGTTCCTTTCCA | 336bp |
|  | LC-R | GAAGGATGCCGAGTAAATCAG |  |
|  | HC-F2 | GAGGAGCAGTACAACAGCAC | 1147bp |
|  | PUR-R | GCACCGTGGGCTTGTACT |  |
| OA-LC/FcH | CMV-F | CTAACAGACTGTTCCTTTCCA | 336bp |
|  | LC-R | GAAGGATGCCGAGTAAATCAG |  |
|  | HC-F2 | GAGGAGCAGTACAACAGCAC | 1147bp |
|  | PUR-R | GCACCGTGGGCTTGTACT |  |
| OA-LC/HCK/FcH  OA-LC/HCK0.5/FcH  OA-LC/HCK/FcH0.5 | CMV-F | CTAACAGACTGTTCCTTTCCA | 336bp |
|  | LC-R | GAAGGATGCCGAGTAAATCAG |  |
|  | HC-F2 | GAGGAGCAGTACAACAGCAC | 1147bp |
|  | PUR-R | GCACCGTGGGCTTGTACT |  |
| OA-LC0.05/HCK/FcH  OA-LC0.10/HCK/FcH  OA-LC0.25/HCK/FcH  OA-LC0.50/HCK/FcH | CMV-F | CTAACAGACTGTTCCTTTCCA | 345bp |
|  | LC-R | GAAGGATGCCGAGTAAATCAG |  |
|  | FcH-F | CAGCCTGAGCTGCGCC | 1147bp |
|  | PUR-R | GCACCGTGGGCTTGTACT |  |
| 2A-FabscFv-Fc | CMV-F | CTAACAGACTGTTCCTTTCCA | 386bp |
|  | HC-R | TCTGCGCTTATAGTGAAACG |  |
|  | LC-F | CGTCACAAAGAGCTTCAACAGG | 713bp |
|  | PUR-R | GCACCGTGGGCTTGTACT |  |
| BsAb-HCK/scFvFcH | CMV-F | CTAACAGACTGTTCCTTTCCA | 520bp |
|  | DHFR-R | GCCTGGTTGATTCATGGC |  |
|  | HC-F2 | GAGGAGCAGTACAACAGCAC | 1147bp |
|  | PUR-R | GCACCGTGGGCTTGTACT |  |
| BsAb-LC/HCK  (also, OA-LC/HCK) | CMV-F | CTAACAGACTGTTCCTTTCCA | 336bp |
|  | LC-R | GAAGGATGCCGAGTAAATCAG |  |
| **Cell line** | **Primer ID** | **Primer sequence (5’-3’)** | **Expected PCR size** |
| BsAb-LC/HCK  (also, OA-LC/HCK) | CHK-F | CACCGTGGACAAGAGCAG | 794bp |
|  | PUR-R | GCACCGTGGGCTTGTACT |  |
| BsAb-LC/HCK/scFvFcH  (also, IRES-FabscFv-Fc)  BsAb-LC/HCK0.5/scFvFcH  BsAb-LC/HCK/scFvFcH0.5  MP-FabscFv-Fc | CMV-F | CTAACAGACTGTTCCTTTCCA | 336bp |
|  | LC-R | GAAGGATGCCGAGTAAATCAG |  |
|  | HC-F2 | GAGGAGCAGTACAACAGCAC | 1147bp |
|  | PUR-R | GCACCGTGGGCTTGTACT |  |
| BsAb-LC0.05/HCK/scFvFcH  BsAb-LC0.10/HCK/scFvFcH  BsAb-LC0.25/HCK/scFvFcH  BsAb-LC0.50/HCK/scFvFcH | CMV-F | CTAACAGACTGTTCCTTTCCA | 345bp |
|  | LC-R | GAAGGATGCCGAGTAAATCAG |  |
|  | HC-F2 | GAGGAGCAGTACAACAGCAC | 1147bp |

**Supplementary Table 3**. List of primers for qRT-PCR

|  | **Gene** | **Forward primer (5’-3’)** | **Reverse primer (5’-3’)** |
| --- | --- | --- | --- |
| 1 | **LC** | GGTCACTATCACCTGCCGTG | GAAGCGAGAAGGGACTCCAG |
| 2 | **HCK** | CGTCAAGGGCCGTTTCACTAT | CCCACCCCATCTGCTACAAT |
| 3 | **scFv-FcH** | GGGACGACTCCAAGAACACC | GGCCCAGTAGCTGATGTAGG |
| 4 | **β-actin** | AGCTGAGAGGGAAATTGTGCG | GCAACGGAACCGCTCATT |

**Supplementary Fig. 1.** Junction PCR performed on gDNA of stably transfected pools to show correct cassette exchange via RMCE. (A) Overview of primer designs across FRT3 and FRT junctions. Primer sequence details used for each stable cell pools were listed in Supplement Table 1. (B) 5’ junction PCR was examined using (CMV-F + DHFR-R) and (CMV-F + LC-R). (C) 3’ junction PCR was examined using (HC-F2 + PUR-R).

**Supplementary Fig. 2.** Junction PCR performed on gDNA of stably transfected pools to show correct cassette exchange via RMCE. (A) Overview of primer designs across FRT3 and FRT junctions. Primer sequence details used for each stable cell pools were listed in Supplement Table 1. (B) 5’ junction PCR was examined using (CMV-F + DHFR-R) and (CMV-F + LC-R). (C) 3’ junction PCR was examined using (HC-F2 + PUR-R) and (CHK-F + PUR-R).

**Supplementary Fig. 3.** Junction PCR performed on gDNA of stably transfected pools to show correct cassette exchange via RMCE. (A) Overview of primer designs across FRT3 and FRT junctions. Primer sequence details used for each stable cell pools were listed in Supplement Table S1. (B) 5’ junction PCR was examined using (CMV-F + HC-R) and (CMV-F + LC-R). (C) 3’ junction PCR was examined using (HC-F2 + PUR-R) and (LC-F + PUR-R).

**Reference**

1. Nguyen,N.T.B., Lin,J., Tay,S.J., Yeo,J. and Khuong,T.N. (2021) Multiplexed engineering glycosyltransferase genes in CHO cells via targeted integration for producing antibodies with diverse complex ‑ type N ‑ glycans. *Sci. Rep.*, 10.1038/s41598-021-92320-x.

2. Merchant,A.M., Zhu,Z., Yuan,J.Q., Goddard,A., Adams,C.W., Presta,L.G. and Carter,P. (1998) An efficient route to human bispecific IgG. *Nat. Biotechnol.*, **16**.

3. Xu,Y., Lee,J., Tran,C., Heibeck,T.H., Wang,W.D., Yang,J., Stafford,R.L., Steiner,A.R., Sato,A.K., Hallam,T.J., *et al.* (2015) Production of bispecific antibodies in ‘knobs-into-holes’ using a cell-free expression system. *MAbs*, **7**, 231–242.

4. Ferreira,J.P., Overton,K.W. and Wang,C.L. (2013) Tuning gene expression with synthetic upstream open reading frames. *Proc. Natl. Acad. Sci. U. S. A.*, **110**, 11284–11289.
